# Geographic atrophy in age-related macular degeneration: phenotypic characterisation for clinical trial consideration

**DOI:** 10.1007/s00417-026-07153-z

**Published:** 2026-02-21

**Authors:** Grace A. Borchert, Peter Charbel Issa, Kanmin Xue, Robert E. MacLaren, Jasmina Cehajic-Kapetanovic, Susan M. Downes, Samantha R. De Silva

**Affiliations:** 1 Nuffield Laboratory of Ophthalmology, Nuffield Department of Clinical Neurosciences, University of Oxford, Oxford, UK; 2 Oxford Eye Hospital, Oxford University NHS Foundation Trust, Oxford, UK; 3 School of Medicine and Health, Department of Ophthalmology, Technical University of Munich, TUM University Hospital, Munich, Germany; 4 Great Ormond Street Hospital for Children NHS Foundation Trust, London, UK

**Keywords:** Non-neovascular age-related macular degeneration, Geographic atrophy, GA AMD, Phenotypes, AI

## Abstract

Geographic atrophy (GA) is an advanced form of age-related macular degeneration (AMD) and a leading cause of central vision loss. Advances in multimodal imaging for GA have improved its phenotypic characterisation, enabling more precise assessment of disease. This is increasingly important for identifying features predictive of progression to inform prognosis and guide patient counselling, enable selection for clinical trials and for disease monitoring both in routine clinical practice and in a research setting. In addition, accurately determining foveal involvement is crucial for selection of patients suitable for emerging therapies. High-resolution imaging is also important to recognise and distinguish GA subtypes such as pachychoroid GA from conventional GA, given their genetic and phenotypic differences and possible variation in response to therapy.

Imaging modalities include colour fundus photography, which is widely available and allows an initial assessment of GA lesions. Fundus autofluorescence imaging permits clear visualisation of GA borders and provides an accurate topographical map of GA pattern and extent, whereas near-infrared reflectance imaging may be superior for evaluation of foveal involvement. Optical coherence tomography (OCT) allows for measurement of the ellipsoid zone which may correlate to visual function and permits differentiation between biomarkers such as nascent GA, incomplete and complete retinal pigment epithelium and outer retinal atrophy (iRORA and cRORA respectively), and identification of pachychoroid GA. Each of these have important prognostic implications and enable accurate selection for clinical trials, monitoring progression and treatment response. Emerging approaches such as red excitation light and high-resolution OCT, may provide more accurate and reliable assessment of atrophic changes. Alongside these advances, artificial intelligence-based tools show great potential in automating GA detection, characterising of structural biomarkers, measuring progression rates and screening patients for clinical trials, increasingly reliability and reproducibility. A better understanding of the important role of multimodal imaging in the classification and assessment of GA, and detection of factors that affect progression will enable clinicians to advise, monitor and, where possible, appropriately treat this major cause of sight loss.

## Key messages

**What is known:**

- Advances in retinal imaging modalities have enabled more precise characterisation of geographic atrophy.
- Assessing features that predict the rate of progression are important for evaluating prognosis, affect selection for clinical trials and potential treatment.

**What is new:**

- We summarise the role, advantages and disadvantages of different imaging modalities for GA assessment and how they can be utilised in clinical practice, for example, to accurately identify patients for potential therapy.
- Newer imaging modalities such as red excitation light AF and high resolution OCT may provide better tolerated and more accurate means of assessment.
- Artificial intelligence holds promising potential to screen geographic atrophy patients for eligibility, quantify structural changes, and assess efficacy in clinical trials.

## Introduction

Geographic atrophy (GA) is one of two forms of advanced age-related macular degeneration (AMD) [1]. It is characterised by confluent areas of photoreceptor, retinal pigment epithelium (RPE) and choriocapillaris loss [2]. Initially, GA may spare the fovea, in which case patients may be less symptomatic with preserved best corrected visual acuity (BCVA). However, when the atrophic area enlarges and affects the fovea, there is a significant decrease in BCVA, affecting reading, driving, and facial recognition. GA may ultimately lead to legal blindness and is one of the most common causes of irreversible vision loss in developed countries.

While there are several highly effective therapies available for neovascular AMD, until recently there have been no approved treatments for geographic atrophy. The United States Food and Drug Administration (FDA) approved two agents in 2023: pegcetacoplan, a complement C3 inhibitor, and avacincaptad pegol, a complement C5 inhibitor, and the Australian Therapeutic Goods Administration (TGA) approved the former in 2025. When administered via intravitreal injection on a monthly or bi-monthly basis, these therapies lead to a reduction in the progression of geographic atrophy on fundus autofluorescence imaging [3–6]. Trials demonstrated significant differences in GA area in treated eyes compared with sham-treated eyes although there was no difference in visual function between groups at 12 and 24 months [7].

**Fig. 1:**
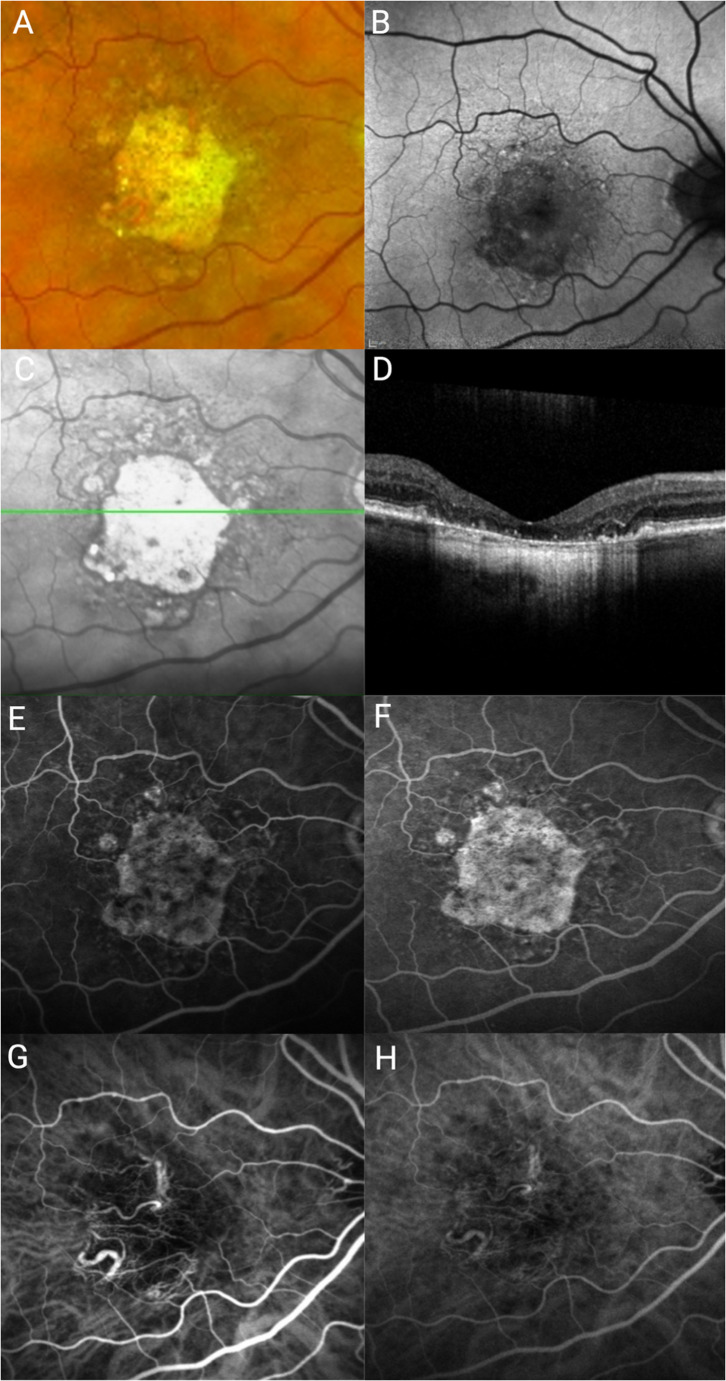
Multimodal imaging of geographic atrophy. (**A**) Pseudocolour fundus photograph (Optos), (**B**) Blue autofluorescence imaging (30-degree image), (**C**) Near-infrared reflectance image (NIR), (**D**) Optical coherence tomography (OCT), **E-F** Fluorescein angiography (early and late phase), **G-H** Indocyanine green angiography (early and mid-phase) in a patient with geographic atrophy

Over recent decades, advances in retinal imaging modalities have enabled anatomical characterisation of GA with increasing resolution and precision (Fig. 1), resulting in standardisation of diagnostic criteria for GA. This allows identification of disease stage, features that predict the rate of progression, stratification of patients for clinical trials and therapy where available, along with subgroup analysis of emerging treatment responses. In particular, accurate assessment of GA and evaluation of foveal involvement are critical, since complement inhibitor therapies are currently recommended for those with established extrafoveal GA. Multimodal imaging also allows monitoring of disease progression and response to treatment. This will also be relevant to the many other potential agents, including genetic therapies for GA that are in clinical trials.

Knowledge of the different modalities of imaging GA, including their advantages and disadvantages is important, since each modality contributes different insights into the retinal structures affected, prognostic factors and pathophysiology of disease. In this review, we describe the important role of multimodal imaging in the classification of GA and detection of factors that affect progression to enable clinicians to advise, monitor and, where possible, appropriately treat this major cause of sight loss.

## Imaging modalities for assessment of geographic atrophy

The classifications of AMD that include GA, and stand-alone classifications of GA according to imaging modality are outlined in Table 1. This has evolved with the development and advances in multimodal imaging. A comparison of the imaging modalities used to assess GA are summarised in Table 2.

**Table 1:** Classifications of AMD that include GA, and stand-alone classifications of GA according to imaging modality, ordered chronologically

Classification	Year	Imaging	Defining criteria
Wisconsin	1991	Colour fundus photograph	AMD Grade 0 to 8 based on drusen size, drusen type, and drusen area
International ARM Epidemiological study group	1995	Colour fundus photograph	Grading drusen 1.1 to 1.6 based on drusen morphology, type, number, size, location, area covered Grading hyperpigmentation and hypopigmentation 0 to 8 Geographic atrophy graded 0 to 8 based on presence, location, area.
Age-Related Eye Disease Study group	2001	Colour fundus photograph	AMD Grade 1 to 4 based on drusen, retinal pigment epithelial pigment abnormalities, geographic atrophy or evidence of neovascular changes.
FAM group	2005	Fundus autofluorescence	Classification of GA into: 1. Focal pattern 2. Banded pattern 3. Patchy pattern 4. Diffuse pattern
Beckman	2013	Colour fundus photograph	AMD classification: Early: medium drusen (63–125 μm) or retinal pigmentary changes (hyper/hypopigmentation) Intermediate: extensive medium drusen and/or at least one large druse (> 125 μm) or GA outside the fovea Late: GA involving the fovea and/or neovascular AMD
Wu group	2014	Optical coherence tomography	Nascent GA describes a subsidence of the outer plexiform layer and inner nuclear layer with a hyporeflective wedge-shaped band
CAM group	2018	Optical coherence tomography	1. iRORA: incomplete RPE and outer retinal atrophy 2. cRORA: complete RPE and outer retinal atrophy
Takahashi group	2018	Optical coherence tomography	Pachychoroid GA is described by features of the pachychoroid phenotype, no drusen and presence of GA.

**Table 2:** Multimodal imaging in geographic atrophy

Imaging modality	Wavelength/ image acquisition (nm)	GA features	Advantages	Limitations	Future Developments
Colour fundus photographs	400–700	Enables visualisation of focal areas of GA, their shape and edges	Accessible in clinic, cost-effective, baseline for monitoring	Inability to detect subtle or early-stage GA, inferior border contrast to FAF	Ultra-widefield, multispectral and hyperspectral colour photography
Fundus auto-fluorescence (FAF)	Blue FAF: 488 Near infrared FAF: 787	GA appears as hypoautofluorescent areas (dark signal) with high border contrast. Can accurately visualise multiple GA foci	Clear identification of GA borders and some prognostic features (GA shape, focality)	Blue FAF: Accurate assessment of foveal involvement can be difficult, media opacity decreases image quality. NIR-FAF: low FAF signal intensity	Fluorescence lifetime ophthalmoscopy FAF using red excitation light [40]
Near-infrared reflectance (NIR)	804–895	GA appears as areas of hyperreflective (bright) signal	Potentially more accurate identification of foveal involvement, image acquisition better tolerated than FAF	Inconsistent contrast due to choroid thickness. Hyperreflective lesions affect border visibility	Ultra-widefield NIR, quantitative analysis of NIR
Optical coherence tomography (OCT)	TD: 830 nm SD-OCT: 800–870 nm SS-OCT: 1050 nm	Absence of RPE most evident as a choroidal hypertransmission defect, loss of ellipsoid zone and photoreceptor layer, thinning of the outer nuclear layer and outer plexiform layer, differentiation of nascent GA, iRORA and cRORA	High-resolution and 3D visualisation of retinal layers allows precise identification of GA lesion size, foveal involvement, factors that affect progression (e.g.RPD) and disease monitoring	More difficult to assess topographical GA size and patterns. Larger GA lesions may not be fully captured due to limited OCT scan field with some devices	Phase contrast OCT (PC-OCT) in GA could show large choroidal vessels and loss of overlying choriocapillaris High-resolution OCT [39]
OCT angiography (OCTA)	840–1060	Choriocapillaris flow deficits in GA	Ability to analyse choriocapillaris, visualisation of concurrent MNV, shorter duration, non-invasive	Motion artefacts may affect image quality, not ubiquitously available	Ultrahigh-speed handheld SS panretinal OCTA
Fundus fluorescein angiography (FA)	465–490	Window defect with early visibility of the choroid	Concurrent MNV may be detected	Invasive	Shift towards non-invasive alternatives, multimodal imaging, portability
Indocyanine green angiography (ICGA)	790–835	GA visualised as a hypofluorescent area	Concurrent MNV may be detected	Invasive	Higher resolution, multimodal imaging

### Colour fundus photography

Colour fundus photographs show GA as a well demarcated area of hypopigmentation secondary to RPE loss with visibility of underlying choroidal vessels (Fig. 1A). It has the advantage of being a simple, accessible and established tool for the initial assessment of GA. Historically, this was the first imaging modality used to visualise GA. In the Wisconsin Age-related Maculopathy grading system reported in 1991, stereoscopic 30° colour fundus photographs were used with a grid to define subfields, and standard circles printed on plastic were used to determine drusen size and area, accompanied by a custom-made lightbox to discriminate drusen [8]. The Wisconsin grading system was also applied to the Beaver Dam Eye study [9].

To further characterise AMD based on colour fundus photographs, the International Age-related maculopathy (ARM) Epidemiological study group classification was developed based on the presence of soft drusen of >63$\:{\upmu\:}\mathrm{m}$, hyperpigmentation and/or hypopigmentation, RPE and associated neurosensory detachment, haemorrhage, GA or fibrous scarring without another vascular cause [10]. Early disease was defined as presence of drusen and RPE pigmentary abnormalities and late disease included GA or neovascular AMD [10]. GA was further classified if present, according to the location and area covered. Similarly, the Age-Related Eye Disease Study (AREDS) system for classification of AMD was also based on standardized stereoscopic 30° colour fundus photographs and detailed in the AREDS Report Number 6. The category of advanced AMD (level 4) included (a) geographic atrophy in the central subfield or (b) evidence of neovascular AMD with fibrovascular or serous pigment epithelial detachment, serous sensory retinal detachment, subretinal pigment epithelial haemorrhage, subretinal fibrosis tissue or photocoagulation for AMD [11]. In the AREDS study, the initial average GA size was 4.3mm^2^ (standard error of the mean 0.24mm^2^) on colour fundus photographs and the overall growth rate was 1.78mm^2^/year [12].

The complexity of previous colour fundus photograph based classification systems was then simplified in the Beckman classification system in 2013 [1]. Normal aging changes were considered to be the presence of small druse or drusen (< 63$\:{\upmu\:}\mathrm{m}$), early AMD to be medium drusen (> 63$\:{\upmu\:}\mathrm{m}$ and <125$\:{\upmu\:}\mathrm{m}$) without pigmentary abnormalities, intermediate AMD with large drusen (> 125$\:{\upmu\:}\mathrm{m}$) or pigmentary abnormalities with at least medium drusen, and late AMD as geographic atrophy or neovascular AMD. This classification is still useful for identifying patients with earlier stages of AMD and predicting prognosis, but is restricted since colour fundus photography has limited sensitivity at the borders of GA and lacks information regarding the retinal layers.

### Fundus autofluorescence imaging

The advent of confocal scanning laser ophthalmoscopy (cSLO)-based fundus autofluorescence (FAF) further facilitated the quantification of GA (Fig. 1B). The most commonly available autofluorescence modality in clinical practice uses incident blue light at 488 nm wavelength which mainly excites fluorophores in lipofuscin (A2E, or N-retinylidene-N-retinylethanolamine). Less commonly, near-infrared light at 787 nm wavelength can be used to stimulate autofluorescence from melanin and melanolipofuscin which are also present in the RPE cells [13]. GA area measurement from blue light FAF images is a widely accepted endpoint for monitoring GA progression and efficacy of therapies in current clinical trials. RPE atrophy results in the loss of lipofuscin-mediated autofluorescence signal, making areas of GA appearing dark on FAF, often with hyperautofluorescent borders. Areas of definitely decreased autofluorescence (DDAF) may also be measured as a clinical trial endpoint. However, these do not capture areas of developing GA, which can still affect visual function and their preservation could help stabilise vision [14]. FAF images are often captured via the Heidelberg Spectralis HRA platform (Heidelberg Engineering, Heidelberg Germany), which incorporates a software tool that automates the measurement of GA area, thus helping to standardise assessment of GA progression in the clinical setting as well as in clinical trials [15]. In a large prospective multicentre observational natural history study, the baseline change in GA size measured on FAF and colour fundus photographs were 0.88mm^2^ and 0.78mm^2^ at 6 months, 1.8mm^2^ and 1.57mm^2^ at 12 months, and 3.14mm^2^ and 3.17mm^2^ at 18 months respectively [16]. From this natural history study, it suggests FAF measurements ‘capture’ a larger size of GA than colour fundus photographs at each time point.

The fundus autofluorescence in age-related macular degeneration (FAM) study classified FAF patterns of GA as none, focal, banded, patchy or diffuse [17] (Table 1). The latter was further subdivided into four groups as reticular, branching, fine granular, fine granular with peripheral punctate spots. An additional pattern was subsequently added, described as trickling [18]. Notably, the trickling subgroup had a higher growth rate (3.02mm^2^/year) compared to other diffuse types (1.67mm^2^/year). However, different patterns on FAF may also represent differential diagnoses of AMD which need to be carefully considered [19].

### Near-infrared reflectance imaging

Near-infrared reflectance imaging (NIR, Fig. 1C) can be simultaneously acquired along with optical coherence tomography (OCT) on the Heidelberg Spectralis platform using a wavelength of 787–820 nm. GA is characterised on NIR images as areas of hyperreflectivity, appearing brighter than non-atrophic areas, since the sclera is highly reflective. Whilst melanin in the RPE also reflects NIR light, it absorbs more than the sclera. NIR can be helpful in identifying lesion boundaries, and foveal involvement of GA may be more accurately evaluated on NIR than FAF because FAF is limited by absorption of blue excitation light by macular pigment [20, 21]. NIR image capture is more comfortable for patients compared to blue-light autofluorescence and is less affected by media opacity (e.g. cataracts) which are common in older patients. NIR is also able to detect reticular pseudodrusen which has prognostic significance for AMD progression [22].

However, a limitation is that NIR can be affected by variations in choroidal thickness, with excellent visualisation of GA in eyes with a thin choroid, but poorer visualisation with a thicker choroid [23]. This may be less of a concern since there is choroidal thinning with age, but is nonetheless a consideration for this modality of imaging. There is currently no consensus classification of GA based on NIR imaging. However, multimodal imaging is important to confirm GA, particularly in questionable cases.

### Optical coherence tomography

OCT provides a highly detailed three-dimensional cross-sectional reconstruction of the retina, which allows evaluation of individual retinal layers in GA, including the photoreceptors, ellipsoid zone, RPE and choriocapillaris (Fig. 1D) [24]. OCT images are acquired using low-coherence interferometry, with the initial time-domain OCT (TD-OCT, 830 nm) scanning sequentially using a moving reference mirror, and more recent spectral-domain OCT (SD-OCT, 800–870 nm) and swept-source OCT (SS-OCT, 1050–1060 nm) using Fourier transformation for quicker and deeper visualisation. On OCT, RPE loss results in choroidal hypertransmission defects due to increased penetration of incident light into the choroid. Ellipsoid zone loss on OCT may be a marker of functional vision loss [25]. Since OCT enables three-dimensional visualisation of vertical degeneration of the outer retina [26], it is increasingly used in clinical assessment and trials involving GA. The DERBY and OAKS phase III clinical trials investigating pegcetacoplan and GATHER study investigating avacincaptad pegol all defined foveal centre point involvement using OCT to classify GA as foveal or extrafoveal [6, 7].

OCT is increasingly used to characterise different stages or patterns of GA. ‘Nascent GA’ has been defined as an early structural change or pre-atrophic disease stage of GA before any RPE loss becomes evident on OCT [27]. It is characterised by thinning and collapse of the outer plexiform layer (OPL) and inner nuclear layer (INL), and hyporeflective wedge-shaped bands in the Henle fibre layer with photoreceptor degeneration. A prospective, longitudinal, observational study demonstrated that nascent GA was a strong predictor for development of GA and proposed that it could become a surrogate endpoint in clinical trials [28].

The Classification of Atrophy Meeting (CAM), through consensus by a panel of international experts [29], developed the currently most widely used OCT-based description of GA. Early atrophic changes were named incomplete RPE and Outer Retinal Atrophy (iRORA) which can progress to complete RPE and Outer Retina Atrophy (cRORA). The disease stage GA is defined as at least one focus of cRORA with a diameter more than 250$\:{\upmu\:}$m. More specifically, the OCT definition of cRORA is defined as (i) a region of hypertransmission of 250$\:{\upmu\:}$m in diameter, (ii) a zone of disruption or attenuation of RPE of at least 250$\:{\upmu\:}$m in diameter, (iii) evidence of overlying photoreceptor degeneration, and (iv) absence of scrolled RPE or other signs of RPE tear.

As well as its importance as a clinical trial endpoint, the differentiation between iRORA and cRORA is key in determining eligibility for GA therapies. The presence of iRORA indicates an elevated risk of progression and therefore potential need for increased monitoring, whereas once extrafoveal cRORA is established then eyes are eligible for GA therapy where available.

There has been significant variability in the interpretation of cRORA by SD-OCT in ‘real world’ applications [30]. Reasons for this include variability in the image quality acquired in real world settings which impacts the ability to distinguish between partial and complete RPE loss, disagreement over the extent of RPE loss, and different levels of experience of graders. The most robust biomarker of GA was found to be choroidal hypertransmission which was associated with the highest agreement between graders [31]. This variability in image quality and grader experience highlights the need for standardised acquisition protocols, training and centralised grading particularly in clinical trials where CAM criteria are an endpoint. AI-based tools may potentially have an important role to improve reproducibility, decrease interobserver variability and better apply the CAM criteria in both clinical trials and clinical practice.

**Fig. 2:**
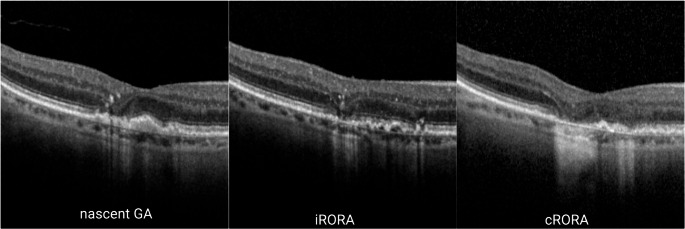
Optical coherence tomography visualisation of nascent GA, iRORA and cRORA

In summary, based on OCT, nascent GA, iRORA and cRORA represent progressive stages of macular degeneration (Table 3; Fig. 2). Nascent GA is an early precursor with intact RPE, while iRORA shows a partial RPE loss and cRORA is characterised by complete RPE loss with photoreceptor degeneration (i.e. established GA).

**Table 3:** Comparison of nascent GA, iRORA and cRORA

Stages on multimodal imaging	Nascent GA	iRORA	cRORA
Stage of progression	Pre-atrophic change	Early stage of atrophy	GA
Appearance on colour fundus photographs	No visible changes	Mild RPE irregularities and depigmentation	Well-defined atrophic area with choroidal vasculature visible
Appearance on FAF	Low or no change	Patchy hypo autofluorescence	Dark hypo autofluorescence
Appearance on NIR	Mildly increased or normal reflectance	Hyperreflective areas representing early atrophy	Well-demarcated hyperreflective area representing RPE loss
RPE on OCT	Intact	Partial loss	Complete loss
Photoreceptors on OCT	Early thinning	Partial loss	Full-thickness loss
Appearance on FFA	No clear leakage or staining	Mild staining	Window defect/ prominent hyperfluorescence

Pachychoroid GA is primarily diagnosed using OCT imaging, particularly with enhanced-depth imaging or swept-source OCT to better visualise the choroid. As a clinical subtype of GA, it is characterised by a thickened choroid and the absence of drusen [32]. There are also changes with dilated Haller’s vessels and attenuation of the choriocapillaris [33]. There are distinct phenotypic and genotypic differences between pachychoroid GA and conventional GA [32]. Pachychoroid GA area has been reported as smaller (0.59mm^2^ vs. 3.76mm^2^; *P* < 0.001), more likely to be unifocal (94.7 vs. 49.6%; *P* < 0.001) and more slowly progressive after adjusting for baseline area and age (0.11 vs. 0.27 mm/year; *P* < 0.001) [34]. Considering the clinical phenotypic and genetic differences of pachychoroid GA, these patients need to be distinguished when designing clinical trials, to ensure that the assessment of potential therapies is not confounded by differences in GA type.

### Retinal angiography

GA can also be visualised by retinal angiography which provides additional insight into underlying choroidal vascular changes and possible complications of GA. Fundus fluorescein angiography (FFA) involves the injection of fluorescein dye into the bloodstream with subsequent imaging of the retina by a fundus camera with excitation wavelength of 488 nm (blue light) and a barrier filter with a wavelength of 500–520 nm (yellow-green light) [35]. Atrophy is visualised as an area of hyperfluorescence due to a window defect caused by loss of RPE (Fig. 1E, F). Indocyanine green angiography (ICGA) uses ICG dye that binds to plasma proteins and allows for visualisation of the choroidal vasculature (Fig. 1G, H). It fluoresces in the near infrared range with an excitation wavelength of 785 nm and barrier filter at 800 nm. It provides important information for visualising the choroidal vasculature since near-infrared light penetrates the RPE, and areas of GA can be visualised although the appearance may not be as marked as in other macular pathologies due to the remaining choriocapillaris in AMD. Both FFA and ICGA are invasive and time-consuming clinical procedures yet they yield little additional information over other imaging modalities for the diagnosis and monitoring of GA, therefore are rarely used for this purpose.

In contrast, OCT angiography (OCTA) is non-invasive and has much faster image acquisition time than conventional angiography. OCTA provides a detailed visualisation of the retinal and choroidal vasculature. It is based on the principle that in a stationary eye, the only movement is related to blood flow and contrast is generated from the difference in movement of cells to the surrounding stationary structures. This difference generates a vascular decorrelation signal which provides visualisation of the retinal and choroidal vasculature. GA on OCTA is demonstrated as a loss of retinal capillaries, and changes in choroidal perfusion seen as a hyporeflective area. The choriocapillaris vascularity index (CVI), the ratio of the vascular area to the total choroidal area, is significantly reduced in geographic atrophy [36]. It has been suggested that CVI could be a predictor of GA progression in a clinical setting [37].

FFA, ICGA and OCTA are however currently most useful in differentiating non-neovascular AMD from neovascular AMD. This is clinically relevant since patients with GA can develop neovascularisation which requires treatment, and there is an increased incidence of MNV in eyes that have been treated with intravitreal injections of complement-inhibitors in the DERBY and OAKS study, as well as real-world outcomes [7, 38].

There are several emerging techniques that could be useful in GA diagnosis and monitoring in the future such as high-resolution OCT, FAF using red excitation light (R-AF), fluorescence lifetime ophthalmoscopy (FLIO) and adaptive optics. High resolution OCT has identified 28 retinal bands which provides further detailed insights into anatomical structural changes and structure-function analysis [39]. Red excitation light AF overcomes the limitations of short wavelength excitation and uncovers features that were previously undetected, with improved comfort and lack of safety concerns for routine clinical monitoring of GA [40]. FLIO is a non-invasive imaging technique that measures the fluorescence lifetime of autofluorescent molecules in the retina [41]. In GA, there is a prolonged fluorescence lifetime because of the loss of RPE and photoreceptors [42] that in a healthy retina would contribute to a shorter fluorescence lifetime. An intermediate lifetime rim can be seen outlining the GA lesion because of the metabolic stress that happens before atrophy occurs [43, 44]. Meanwhile, adaptive optics (AO) can give a high-resolution visualisation of the photoreceptors, RPE and microvasculature [45, 46]. AO could have a role in tracking cellular changes at high resolution with dark hyporeflective areas demonstrating photoreceptor and RPE loss in GA, and at the border a mix of normal and damaged cells.

## Phenotypic patterns predicting geographic atrophy progression

Features that predict GA progression are important to inform our understanding of pathophysiology, counsel patients and help stratify which GA patients would benefit from emerging therapies. Previous studies have reported GA progression rates for total study populations at between 0.53 to 2.6mm^2^/year [47, 48]. The rate of progression is multifactorial and is affected by features of the lesion (such as size, shape, focality, location and FAF phenotype), presence of reticular pseudodrusen (RPD), whether the fellow eye is affected, and genetic, environmental and demographic factors.

### GA size

**Fig. 3:**
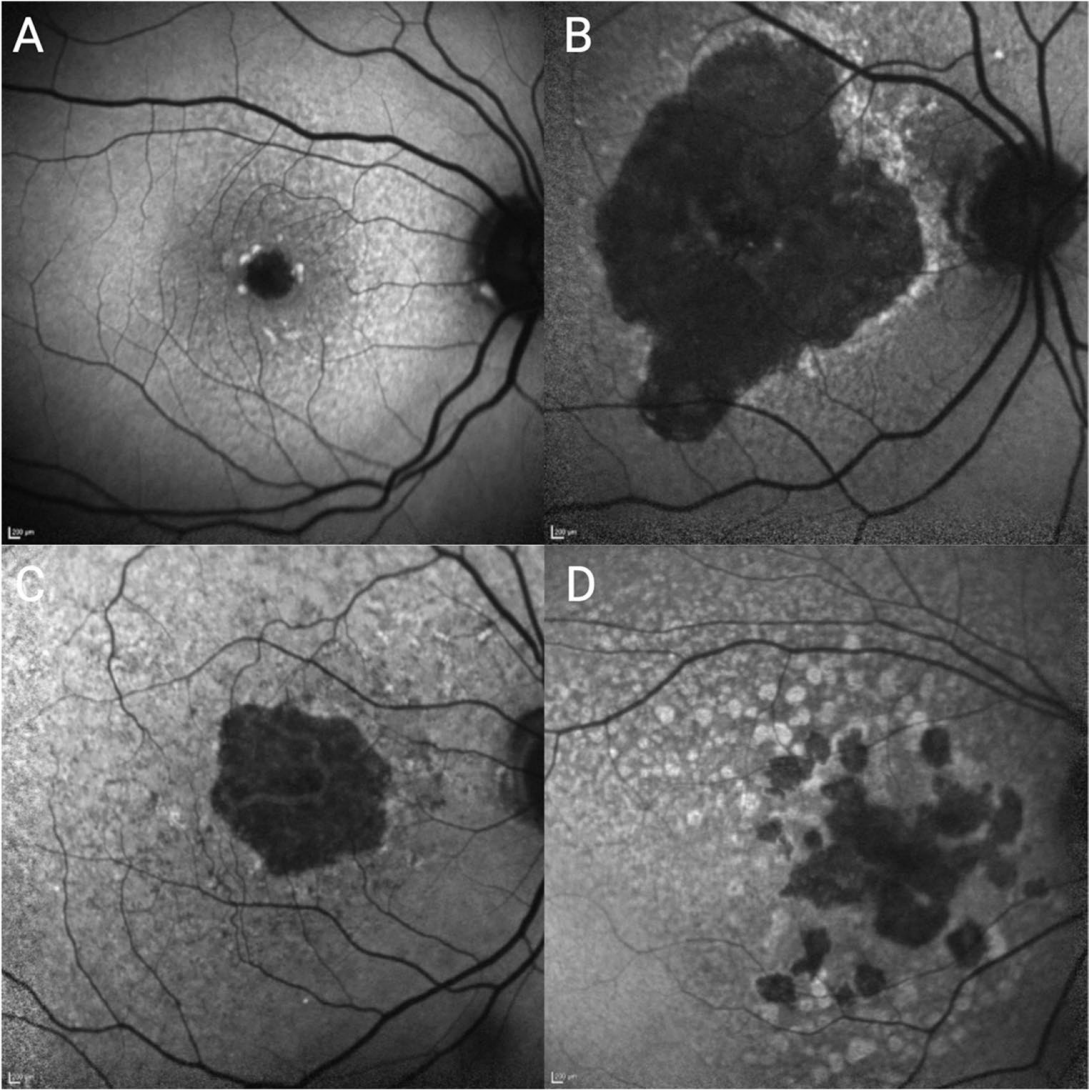
Features associated with GA progression based on blue fundus autofluorescence. Lesion size in fovea-involving GA: small (**A**) versus large (**B**) area. Focality: unifocal (**C**) compared to multifocal (**D**) GA involving the fovea

Studies have demonstrated that GA lesions of smaller area at baseline show a slower rate of progression over time (Fig. 3A, B) [12]. In the FAM study, the median progression rate of GA with baseline of < 1 disc area (defined as > 1.33mm^2^) was 0.74mm^2^/year which was less than eyes with a larger baseline GA area (5–10 disc area) that had a progression rate of 1.88mm^2^/year.

### GA focality

Multifocal GA lesions expand at a greater rate compared to unifocal lesions (Fig. 3C, D) [16]. In a natural history study of 307 participants with multifocal GA and 106 participants with unifocal GA, the growth rate was significantly greater in eyes with multifocal lesions. At 12 months, multifocal lesions had a growth rate of 1.97 mm^2^/year versus 1.05 mm^2^/year in the unifocal group [16]. The Beaver Dam Eye study described that with computer-assisted measurements of digital images, multifocal lesions were more likely to increase in atrophy (2.4mm^2^/year) compared to unifocal lesions (0.4mm^2^/year) [49]. Variations in rate may be attributed to different methods of measurement, baseline size, and features such as RPD. A geographic atrophy circularity index (GACI) has been developed and found eyes with individual lesions that deviated most from a circle were more likely to be related to multifocal disease and have a higher rate of progression [50].

### Fellow eye and laterality

Geographic atrophy in one eye is a strong predictor of development of GA in the fellow eye [12]. There is a high concordance between the enlargement rates of GA between the two eyes of patients with bilateral GA (correlation coefficient was 0.76). Notably, the AREDS study only recruited participants with unilateral GA, while the FAM study recruited both unilateral and bilateral GA participants [51]. It was estimated in AREDS that the average time from progressing from unilateral to bilateral GA was 7 years [12]. In the AREDS2 study, it was demonstrated that GA growth was significantly faster in patients who had bilateral GA relative to those who had unilateral GA (square root rate at 0.31 mm/year vs. 0.23 mm/year, respectively (*p* < 0.0001).

### Foveal involvement

Extrafoveal geographic lesions progress more rapidly compared to foveal lesions [16]. Foveal lesions, defined as the presence of GA within a 300$\:{\upmu\:}\mathrm{m}$ diameter circle centered on the fovea, progressed at a growth rate of 1.28 mm^2^/year compared to extrafoveal lesions at a rate of 2.05mm^2^/year (*p* = 0.001) [16]. On FAF, a directional kinetics analysis in patients with baseline foveal sparing geographic atrophy suggested that lesions progressed toward the fovea more slowly compared to progression towards the periphery [16]. On OCT analysis, predictive features for the development of atrophy at the fovea included foveal outer retinal thickness, distance from the foveal centre and foveal thin double layer sign, characterised by a thin hyporeflective space between RPE and Bruch’s membrane as a result of collapse of choriocapillaris [52]. Appearance of the latter predicted development of atrophy at the centre of the fovea at 2 years in eyes with extrafoveal GA at baseline with an odds ratio of 0.138 (*p* = 0.017) in their multivariate logistic regression analysis [52].

### Features on OCT

OCT biomarkers allow identification of structural changes that predict GA progression such as reticular pseudodrusen (RPD), nascent GA and iRORA. RPD also called subretinal drusenoid deposits, located above the RPE in the subretinal space are strongly associated with GA development and progression [53], and are also associated with the development of multifocal GA [54, 55]. Hyperreflective foci in the outer retina have also been described and represent migrating RPE cells preceding GA development. Nascent GA and iRORA are may evolve into cRORA or established GA [24, 56]. Choroidal hypertransmission is a primary biomarker for diagnosing GA and monitoring progression in clinical trials as the hypertransmission size correlates with GA size [57, 58]. A recent study investigated the relationship between outer retinal tubulations and GA lesion growth rate [59]. In 300 patients with geographic atrophy who were part of the OAKS and DERBY trials, 32% (96 out of 300 GA patients) had an outer retinal tubulation at baseline [59]. A fully formed outer retinal tubulation at baseline was associated with a slower GA growth rate compared to patients without an outer retinal tubulation (1.52mm^2^/year vs. 2.13mm^2^/year). This may have relevance for future clinical trial design.

## GA phenotypes in clinical trial and endpoints on multi-modal imaging

As of April 2025, there were 37 interventional clinical trials for GA registered on ClinicalTrials.gov that were recruiting, active or completed. Of these, 22 clinical trials registered as phase II-IV assessed GA area using FAF imaging (Table 4). Five trials included patients with only extra-foveal GA (22.7%), two trials included both foveal-involving and extra-foveal GA (9.1%), and one trial included only foveal-involving GA (4.6%). The remainder were 14 trials that did not specify whether patients with foveal or extra-foveal GA were to be recruited in the inclusion criteria. The baseline GA area for inclusion was broad, ranging from 0.5mm^2^ to 17.76mm^2^.

**Table 4:** Interventional clinical trials for GA detailing intervention, imaging modality for assessment, and inclusion criteria based on GA characteristics and visual acuity. For comparison purposes, when the GA size was reported as disc areas this was converted to area in mm^2^ based on one disc area being 2.89mm^2^. Foveal = geographic atrophy affecting the fovea; extra-foveal = geographic atrophy not involving the fovea . NS = not specified, CFP = colour fundus photography, OCT = optical coherence tomography, FAF = fundus autofluorescence imaging

NCT Number	Sponsor	Phase	Study Status	Intervention	Imaging Modality	Foveal	Extra-foveal	Unifocal	Multifocal	Size (mm2)	VA
											(letters ETDRS)
NCT04770545	Apellis	3	Active	PEGCETACOPLAN (APL-2)	FAF	NS	NS	yes	> 1 focal lesion > 1.25mm2	> 2.5 and < 17.5	> 24
NCT06722157	Boehringer Ingelheim	2	Recruiting	Pegcetacoplan	FAF	no	yes	NS	NS	> 2.5 and < 17.5	> 24
NCT05019521	Alexion	2	Completed	Danicopan	FAF, CFP, SD-OCT	no	yes	yes	> 1 focal lesion > 0.5mm2	0.5 to 17.76	84 to 24
NCT06635148	Janssen Research & Development	2	Recruiting	JNJ-81,201,887	FAF, CFP, SD-OCT	yes	no	yes	> 1 focal lesion > 1.27mm2	> 1.27	< 35
NCT05893537	Cognition Therapeutics	2	Active	CT1812	FAF	NS	NS	NS	NS	NS	> 24
NCT02686658	IVERIC bio	2/3	Completed	Avacincaptad Pegol	FAF	NS	NS	yes	> 1 focal lesion > 1.25mm2	> 2.5 and < 17.5	24 to 83
NCT03815825	Ionis	2	Completed	IONIS-FB-LRx	FAF	NS	NS	yes	> 1 focal lesion > 1.25mm2	> 2.5 and < 17.5	> 35
NCT03845582	Alkeus	3	Completed	ALK-001	NS	NS	NS	NS	NS	NS	
NCT05811351	Janssen Research & Development	2	Active	JNJ-81,201,887	FAF	no	yes	yes	> 1 focal lesion > 1.27mm2	> 1.27	< 35
NCT04435366	IVERIC bio	3	Completed	Avacincaptad Pegol	FAF	no	yes	NS	NS	NS	24 to 83
NCT01782989	Paul Yates	2/3	Completed	ORACEA	FAF	NS	NS	NS	NS	> 1.45 to < 20.23	20 to 85
NCT05626114	Genentech	2	RECRUITING	OpRegen	OCT	NS	NS	NS	NS	NS	> 29
NCT01342926	GlaxoSmithKline	2	Completed	GSK933776	CFP, FAF	NS	NS	NS	NS	1.9 to 17	> 35
NCT06541704	Regeneron	3	Recruiting	Pozelimab, Cemdisiran	FAF	no	yes	NS	NS	> 2.5 and < 17.5	> 35
NCT05839041	Aviceda Therapeutics	2	Active	AVD-104	FAF	yes	yes	NS	> 1 focal lesion > 1.25mm2	> 1.25	5 to 55
NCT03525600	Apellis	3	Completed	APL-2	FAF	NS	NS	yes	> 1 focal lesion > 1.25mm2	> 7.23	5 to 35
NCT03525613	Apellis	3	Completed	APL-2	FAF	NS	NS	yes	> 1 focal lesion > 1.25mm2	> 2.5 and < 17.5	> 24
NCT04465955	NGM Biopharmaceuticals	2	Completed	NGM621	FAF	NS	NS	yes	> 1 focal lesion > 1.25mm2	> 2.5 and < 17.5	> 34
NCT06510816	Annexon	3	Recruiting	ANX007	NS	NS	NS	NS	NS	NS	NS
NCT04656561	Annexon	2	Completed	ANX007	FAF	NS	NS	NS	> 1 focal lesion > 1.25mm2	> 2.5 to < 17.5	24 to 83
NCT02659098	Janssen Research & Development	2	Completed	CNTO 2476 3.0 × 10^5 cells	FAF	NS	NS	NS	NS	> 1.25	5 to 55
NCT06659549	Galimedix Therapeutics	2	Recruiting	GAL-101	FAF	no	yes	yes	> 1 focal lesion > 1.25mm2	1.25 to 12	> 50

Predictive factors for GA progression need to be carefully considered in the design of clinical trials, taking into account the natural history of each GA phenotype, when evaluating treatments with appropriate selection of patients. For example, smaller GA lesions that are foveal-involving and unifocal tend to be slower to progress, which means that a longer clinical trial duration would be required to assess treatment efficacy in such GA cohorts. Meanwhile, GA with higher predictive factors for progression may have potential to demonstrate treatment effects more quickly.

## Future directions – artificial intelligence in clinical trials for GA

Artificial intelligence (AI) models provide the possibility of an efficient, automated, and reproducible approach to screening GA patients at scale for eligibility, facilitate automated GA area measurements and OCT segmentation, and evaluate functional endpoints for treatment efficacy in clinical trials. Further research is needed to improve the robustness of AI in real-world practice to ensure that it performs well with routinely collected multimodal images of variable quality, on different devices and using a range of protocols. There is also a need for robust governance framework to ensure the ethical integration of AI into clinical trials for GA.

A deep learning model has been trained on OCT scans to identify potentially eligible patients for the HORIZON trial [60]. This AI system shortlisted a significant number of potentially eligible GA patients with higher precision compared to traditional keyword based electronic health record search (1139 patients vs. 693) [60]. A limitation of this is that FAF and OCT imaging have inherent differences that may affect the total GA area. It has been suggested that the AI system underestimates the area of GA for areas > 17.5mm^2^ on FAF.

There is a need to standardise GA area measurements and OCT segmentation to monitor progression. Automated OCT monitoring using deep learning-based algorithms has been compared to FAF measurements in the phase III OAKS and DERBY trials for pegcetacoplan. It was demonstrated that there was a high correlation between manual FAF and automated OCT-based RPE measurements [61–63]. Similar findings were also reported by another retrospective study comparing definitely decreased autofluorescence on FAF with automated segmentation and en-face OCT [64]. The GA Monitor (RetInSight, Vienna, Austria) incorporates AI algorithms that segment and quantify RPE and EZ loss and thickness which is a tool that has EU Medical Device Regulation approval. Real-world data will be needed to evaluate its effectiveness in clinical deployment and studies.

Evaluating visual function in GA patients is also very important for assessing treatment effects. Different machine-learning approaches (random forest, LASSO regression and multivariate adaptive regression splines) have been assessed in predicting retinal sensitivity in patients recruited for the OMEGA study [65]. The random forest model from these models demonstrated the highest accuracy in predicting retinal sensitivity in GA patients [65]. However, further work is needed to validate this in a larger cohort and explore the integration of this as a functional surrogate endpoint for GA clinical trials.

## Conclusion

Defining GA as a single entity needs to be carefully reconsidered given the complexity and heterogeneity of patient phenotypes. Reflecting on the historical classifications of GA provides helpful guidance for reclassifications moving forward because there is a need for a precise, natural history-driven, and efficient multimodal approach to stratify GA for the purpose of clinical assessment and clinical trials.

**Fig. 4:**
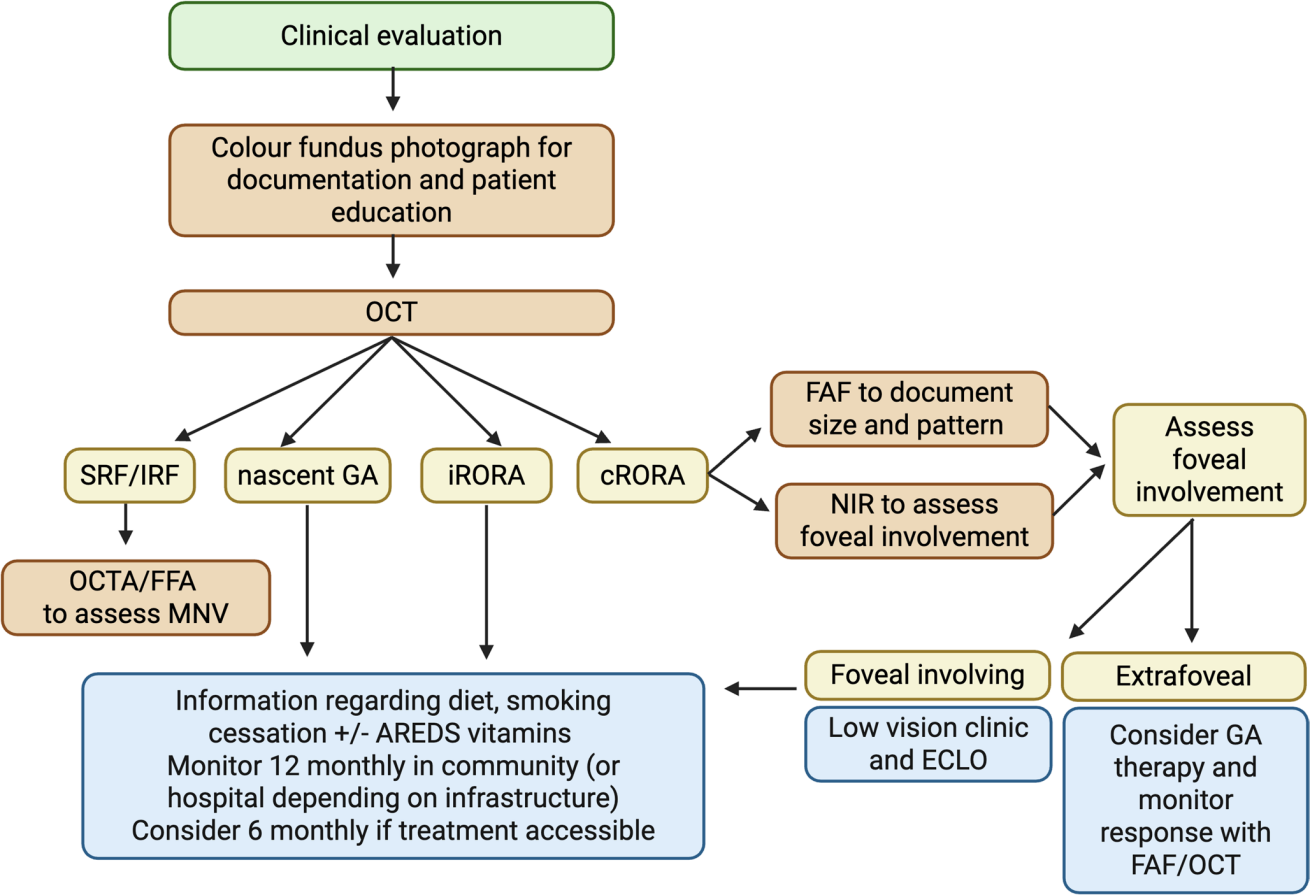
Workflow for the assessment of GA using available imaging modalities. Imaging modalities in red, clinical features in yellow and management in blue. Foveal = geographic atrophy affecting the fovea; and extra-foveal = geographic atrophy not involving the fovea. CFP = colour fundus photography, OCT = optical coherence tomography, OCTA = optical coherence tomography angiography, FAF = fundus autofluorescence imaging and NIR = near infrared reflectance

In a clinical trial setting FAF enables sensitive detection and delineation of GA and OCT allows identification of nascent GA, iRORA and cRORA to allow for precise assessment of structural biomarkers. Foveal involvement may be assessed on NIR as a complementary tool whereas OCTA can be considered an adjunct modality for characterising choriocapillaris changes and neovascularisation. In routine clinical practice, GA imaging depends on equipment availability and resources in a busy clinical setting, and whether therapy is available if indicated. We propose a practical workflow combining the available imaging modalities that clinicians could use for the assessment of GA, should time and facilities allow (Fig. 4).

In the future, developments in multimodal imaging including high resolution OCT and red excitation light AF will be helpful to more clearly and consistently capture the granularity of geographic atrophy. In parallel, particularly relevant to emerging clinical trial development, outcome measures such as microperimetry to assess retinal sensitivity need to be incorporated in this process. AI holds great potential to screen GA patients for eligibility, quantify structural changes in GA, and assess efficacy in clinical trials. With emerging therapeutic approaches, further work is urgently needed to better diagnose, monitor and manage GA.

## Data Availability

No datasets were generated or analysed during the current study.
